# Tet1 Suppresses p21 to Ensure Proper Cell Cycle Progression in Embryonic Stem Cells

**DOI:** 10.3390/cells11081366

**Published:** 2022-04-17

**Authors:** Stephanie Chrysanthou, Julio C. Flores, Meelad M. Dawlaty

**Affiliations:** 1 Ruth L. and David S. Gottesman Institute for Stem Cell and Regenerative Medicine Research, Albert Einstein College of Medicine, 1301 Morris Park Ave, Bronx, NY 10461, USA; stephaniechrys24@gmail.com (S.C.); julio.flores@einsteinmed.edu (J.C.F.); 2 Department of Genetics, Albert Einstein College of Medicine, 1301 Morris Park Ave, Bronx, NY 10461, USA; 3 Department of Developmental & Molecular Biology, Albert Einstein College of Medicine, 1300 Morris Park Ave, Bronx, NY 10461, USA

**Keywords:** Tet1, ESC, cell cycle, p21, proliferation

## Abstract

Ten eleven translocation 1 (Tet1) is a DNA dioxygenase that promotes DNA demethylation by oxidizing 5-methylcytosine. It can also partner with chromatin-activating and repressive complexes to regulate gene expressions independent of its enzymatic activity. Tet1 is highly expressed in embryonic stem cells (ESCs) and regulates pluripotency and differentiation. However, its roles in ESC cell cycle progression and proliferation have not been investigated. Using a series of Tet1 catalytic mutant (*Tet1^m^*^/*m*^), knockout (*Tet1*^−/−^*)* and wild type (*Tet1*^+/+^) mouse ESCs (mESCs), we identified a non-catalytic role of Tet1 in the proper cell cycle progression and proliferation of mESCs. *Tet1*^−/−^, but not *Tet1^m^*^/*m*^, mESCs exhibited a significant reduction in proliferation and delayed progression through G1. We found that the cyclin-dependent kinase inhibitor p21/*Cdkn1a* was uniquely upregulated in *Tet1*^−/−^ mESCs and its knockdown corrected the slow proliferation and delayed G1 progression. Mechanistically, we found that p21 was a direct target of Tet1. Tet1 occupancy at the p21 promoter overlapped with the repressive histone mark H3K27me3 as well as with the H3K27 trimethyl transferase PRC2 component Ezh2. A loss of Tet1, but not loss of its catalytic activity, significantly reduced the enrichment of Ezh2 and H3K27 trimethylation at the p21 promoter without affecting the DNA methylation levels. We also found that the proliferation defects of *Tet1*^−/−^ mESCs were independent of their differentiation defects. Together, these findings established a non-catalytic role for Tet1 in suppressing p21 in mESCs to ensure a rapid G1-to-S progression, which is a key hallmark of ESC proliferation. It also established Tet1 as an epigenetic regulator of ESC proliferation in addition to its previously defined roles in ESC pluripotency and differentiation.

## 1. Introduction

The ten eleven translocation (Tet) family of proteins (Tet1/2/3) are epigenetic modifiers that promote DNA demethylation through the iterative oxidation of 5-methylcytosine (5mC) to 5-hydroxymethylcytosine (5hmC) and other derivatives [1,2,3]. They can also form complexes with other epigenetic modifiers and transcription factors to regulate gene expressions independent of their enzymatic activity in DNA demethylation [4,5,6]. Tet enzymes are dynamically expressed during development and in various embryonic and somatic cell types. In embryonic stem cells (ESCs), Tet1 is a key regulator of pluripotency where its enzymatic activity is required for the proper demethylation of pluripotency genes [2,7] and its non-enzymatic functions promote the stable repression of lineage specifiers [4,5,6]. The biological relevance of Tet1 in ESC self-renewal and pluripotency is well-investigated in the field, but very little is known about the role of Tet1 in regulating ESC proliferation. 

A key feature of ESCs is their rapid proliferation, which is marked by a unique cell cycle structure that has an extended DNA synthesis (S) phase and very short Gap (G1/G2) phases [8]. Mouse ESCs (mESCs) progress through the cell cycle in as fast as 6 h and the S phase constitutes ~65% of the cell cycle [9]. mESCs maintain this cell cycle structure until they differentiate [9,10]. This distinct cell cycle of mESCs is mainly driven by the high expression of cyclins, increased cyclin-dependent kinase (Cdk) activity and the absence of Cdk inhibitors (CKIs) [8,9]. As such, the typical oscillatory activity of Cyclin-Cdk complexes present in a somatic cell cycle is absent in mESCs. For example, Cyclin E/Cdk2 and Cyclin A/Cdk2 activities are so high throughout the ESC cell cycle that they are considered to be cell cycle-independent. A high Cdk2 activity is essential for a rapid G1 phase progression and the establishment of the unique ESC cycle structure [11]. p21/*Cdkn1a* is a key CKI that inhibits Cdk2 and is a negative regulator of the G1/S transition. Therefore, it is suppressed in ESCs to allow for a rapid progression through G1 [8,9,10]. However, the mechanisms by which p21 is repressed in ESCs have not been well-defined. Although it is widely established that p21 is a downstream target of p53, which can readily activate p21, the p53-independent regulation of p21 has also been reported. For example, in human ESCs, it has been shown that the p21 expression is epigenetically silenced through the deposition of the repressive histone mark H3K27me3 at the p21 promoter, which prevents p21 activation even in the presence of p53 [12]. This suggests that the epigenetic modifications of the p21 promoter override the transcriptional activation by p53 and serve as a safeguard mechanism for proper cell cycle progression.

Recently, we and others have implicated Tet1 in H3K27 trimethylation and the suppression of developmental genes in mESCs. Tet1, independent of its enzymatic activity, facilitates the recruitment of the histone trimethyltransferase PRC2 to deposit H3K27me3 and establish a bivalent state in developmental genes [4,6]. This silences the developmental programs in mESCs, but keeps them poised for activation upon differentiation and is, therefore, important in maintaining the pluripotent state. However, it is not known whether Tet1 can also regulate the cell cycle and proliferation of mESCs through histone modifications and/or DNA demethylation. In this study, we report a catalytic independent role of Tet1 in the regulation of the mESC cell cycle progression through the suppression of p21. We found that Tet1 directly binds to the p21 promoter and, independent of any DNA demethylation activity, recruits PRC2 for H3K27 trimethylation to repress p21. This allows for a rapid G1-to-S progression in mESCs. Consistently, Tet1 knockout, but not catalytic-deficient mESCs, had an extended G1 phase, an increased p21 expression and reduced levels of PRC2 and H3K27me3 at its promoter. The knockdown of p21 or the re-expression of catalytic-dead Tet1 in Tet1 knockout mESCs rescued the cell cycle and proliferation defects. Together, our findings identified Tet1 as an epigenetic regulator of ESC cell cycle progression and proliferation.

## 2. Materials and Methods

### 2.1. Embryonic Stem Cell Culture and Proliferation Assays

*Tet1*^−/−^ and *Tet1^m^*^/*m*^ mESCs were previously generated in our lab [6]. All mESC lines tested negative for mycoplasma by a PCR test and were cultured onto irradiated feeders in a media-containing serum/LIF (DMEM supplemented with 10% FBS, 2 mM glutamine, 1 × non-essential amino acids, 100 U/mL penicillin, 100 μg/mL streptomycin, 0.02 ug/mL LIF, 50 mM b-mercaptoethanol). The cells were passaged onto feeder-coated plates once they reached 70–80% confluency. For RNA and DNA extraction, the mESCs were pre-plated onto gelatin to remove the feeders and then seeded onto gelatin overnight before harvesting. For the differentiation assay, pre-plated mESCs were seeded onto gelatin in mESC media for 12 h and then cultured in mESC differentiation media (without LIF and supplemented with 1 μM retinoic acid) for 3 d. For the proliferation assays, 25,000 cells of each clone were seeded onto gelatin in 12-well plates in triplicate. The viable cells were counted using trypan blue and hemocytometer each day for 4 d. A two-way ANOVA test was used to calculate the statistical significance.

### 2.2. Cell Cycle Analysis and Apoptosis Assays

The mESCs were cultured onto gelatin and on the following day they were arrested in G2/M by a Nocodazole treatment (M1404, Sigma-Aldrich, Inc., St. Louis, MO, USA, 100 ng/mL for 16 h). The cells were released from the arrest by removing the Nocodazole-treated media, washing with 1× PBS twice and culturing in fresh mESC media for 2, 4, 5 and 7 h. The cells were fixed in 70% ethanol/PBS followed by staining for 30 min in PBS containing 0.1 mg/mL RNase A and 50 μg/mL propidium iodide (PI) (P4864, Sigma-Aldrich, Inc., St. Louis, MO, USA). The cell cycle analysis was performed using a BD LSR II flow cytometer (BD, Biosciences, Franklin Lakes, NJ, USA) and FlowJo software (v.10.8.0). For the quantification of the apoptotic cells, the ESCs were cultured onto gelatin for 24 h and stained with Annexin V and 7AAD using an Annexin V kit (556547, BD Biosciences, Franklin Lakes, NJ, USA) following the manufacturer’s guidelines as previously described [13]. Annexin V staining was analyzed on a BD LSR II flow cytometer using FlowJo software (v.10.8.0). Early (7AAD^−^ Annexin V^+^) and late (7AAD^+^ Annexin V^+^) apoptotic cells were quantified and plotted.

### 2.3. RT-qPCR and ChIP-qPCR

A total of 2 μg of RNA, extracted from feeder-free mESCs by an Omega E.Z.N.A Total RNA kit (Omega Bio-tek, Inc., Norcross, GA, USA), was used to synthesize cDNA using a Superscript III First-Strand synthesis system (Invitrogen, Waltham, MA, USA). A real-time quantitative PCR was performed using a SYBR green master mix (Applied Biosystems, Waltham, MA, USA) in a QuantStudio 6 Flex Real-Time PCR system following the standard protocols using previously published primers [14,15,16,17,18,19,20,21] as listed in Table S1. The relative gene expression level was analyzed by a comparative Ct method and was normalized to *Gapdh*. A two-way ANOVA test was used to calculate the statistical significance between the three groups. ChIP experiments were performed using feeder-free mESCs cultured onto gelatin following previously published protocols [22] using antibodies against Tet1 (GTX125888, GeneTex, Irvine, CA, USA), Ezh2 (CST 5246, Cell Signaling Technologies, Danvers, MA, USA), and H3K27me3 (07449, Millipore, Burlington, MA, USA). The DNA concentration was measured using a Qubit 2.0 Fluorometer (Invitrogen, Waltham, MA, USA). The protein enrichment at specific loci was quantified by qPCR as mentioned above using the primers in Table S1. ChIP-qPCR signals were calculated as a fold enrichment using a 10% input and IgG as the control. In the case of the Tet1 ChIP-qPCR, the enrichment was also normalized over the *Tet1*^−/−^ controls.

### 2.4. Western Blotting

The cells were lysed in a Radioimmunoprecipitation assay (RIPA) buffer (50 mM Tris-HCl, pH 7.4, 250 mM NaCl, 2% Nonidet-P40, 2.5 mM EDTA, 0.1% SDS, 0.5% DOC Sigma-Aldrich, Inc., St. Louis, MO, USA) supplemented with PIC and PMSF. The lysates were resolved on an 8–12% SDS-PAGE (Mini-PROTEAN electrophoresis chamber, Bio-Rad, Hercules, CA, USA) and transferred onto PVDF membranes (Mini Trans-Blot apparatus, Bio-Rad, Hercules, CA, USA) following the manufacturer’s protocols. The membranes were blocked in 5% milk in PBS with 0.1% Tween-20 (PBST) and incubated overnight at 4 °C or for 1 h at room temperature with primary antibodies (p21: BD Pharmingen 556431; p53: CST2524T; p27: Santa Cruz sc1641; CycD1: CST2978T; Cdk1: Abcam ab18; Cdk2: CST2546T; β-actin: Abcam ab6276). Secondary antibody incubations (HRP-anti-mouse Calbiochem 401253 or HRP-anti-rabbit Calbiochem 401393, 1:3000) were carried out for 1 h at room temperature. In all experiments, β-actin was used as the loading control. The quantification of the Western blot band signal intensities was performed using Image J (v.1.53k) and the data were normalized to the respective actin signal intensities and then plotted.

### 2.5. Lentivirus Preparation for p21 Knockdown

HEK293T cells were cultured in 10% FBS Dulbecco’s Modified Eagle Medium (DMEM) and transfected with a 10 µg pFUGW-H1 empty vector or pFUGW-H1 p21 shRNA1 (Addgene Watertown, MA, USA, plasmid # 25868) [23] along with lentiviral packaging plasmids pPAX2 (7.5 µg) and pMDG (2.5 µg) using an Xtremegene Transfection reagent (06365787001, Roche, Basel, Switzerland). The media were changed after 16 h. The following day, the lentivirus-containing supernatant was collected at 24 h and at 48 h and concentrated by a Lenti-X Concentrator (631232,Takara, Tokyo, Japan), according to the manufacturer’s protocol. The mESCs cultured onto the gelatin at a 50% confluency were transduced with a pFUGW-H1 empty vector or a pFUGW-H1 p21 shRNA1 lentivirus supplemented with polybrene. The GFP-positive cells were sorted by flow cytometry and expanded in the culture for use in the experiments.

### 2.6. Bioinformatic Analysis

The DNA methylation levels and the enrichment of Tet1, H3K27me3 and H3K4me3 peaks at the promoter of p21 were visualized in the integrated genome browser (IGV) using our previously published Tet1 CUT&Tag datasets in *Tet1*^+/+^ and *Tet1^m^*^/*m*^ mESCs, H3K4me3 and H3K27me3 CUT&Tag datasets and WGBS datasets in *Tet1*^+/+^, *Tet1^m^*^/*m*^ and *Tet1*^−/−^ mESCs (GSE176389) [6]. The genes implicated in the cell cycle progression were identified using our previously published RNA-seq datasets in *Tet1*^+/+^, *Tet1^m^*^/*m*^ and *Tet1*^−/−^ ESCs (GSE176389) [6] and their expression was plotted as a heatmap using the pheatmap package in R software (v. 4.1.0).

## 3. Results

### 3.1. Deficiency of Tet1, but Not of Its Catalytic Activity, Leads to Reduced Proliferation and Extended G1 Phase in mESCs

**Figure 1:**
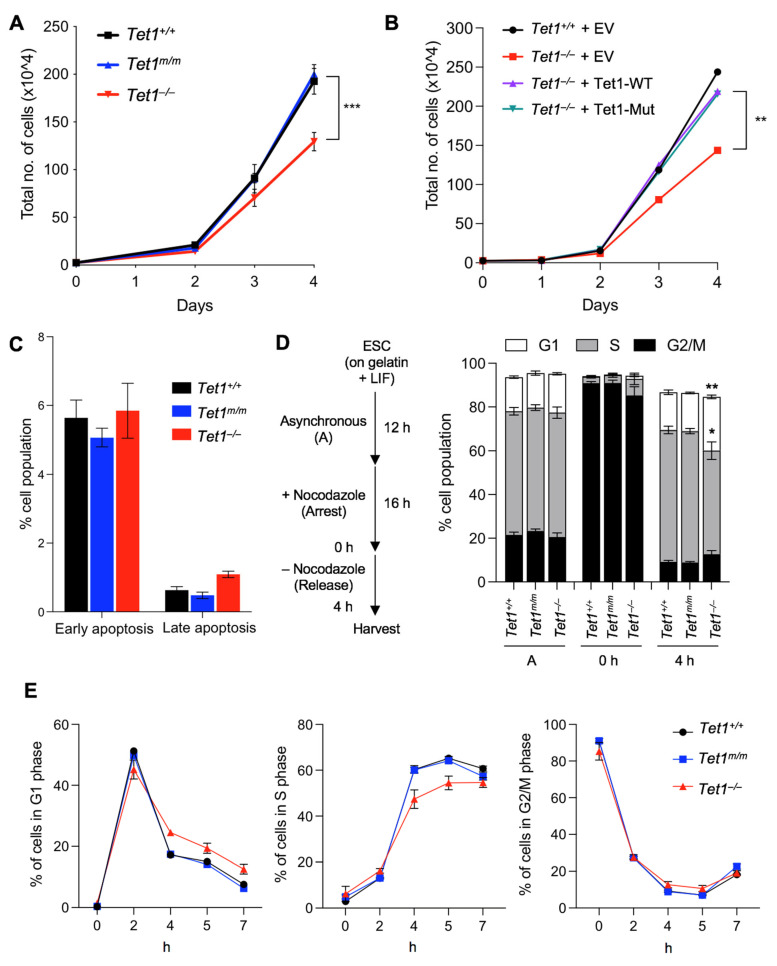
Loss of Tet1, but not the loss of its catalytic activity, reduces mESC proliferation and delays G1/S transition. (**A**) Growth curve of mESCs of indicated genotypes over a 4–day period. Three lines of each genotype were used. (**B**) Growth curve of *Tet1*^−/−^ mESCs stably overexpressing wild type (WT) or catalytic mutant (Mut) Tet1 transgene. *Tet1*^+/+^ and *Tet1*^−/−^ mESCs transfected with an empty vector were used as controls. (**C**) Quantification of early (7AAD^−^ Annexin V^+^) and late (7AAD^+^ Annexin V^+^) apoptotic cells by flow cytometry in mESCs of indicated genotypes. Three lines of each genotype were used. Note that the number of apoptotic cells was not increased in *Tet1*^−/−^ mESCs. (**D**) Schematic of cell cycle synchronization strategy (**left**) and percentage of mESCs in each phase of the cell cycle 4 h after release from Nocodazole block (**right**). Three lines of each genotype were used. (**E**) The percentage of mESCs in each phase of the cell cycle at the indicated timepoints shown on the x-axis after release from Nocodazole. Three independent mESC lines of each genotype were used. In all panels, data are presented as ± SEM; statistically significant *** *p* < 0.001, ** *p* < 0.01 and * *p* < 0.05 (two-way ANOVA with Holm–Sidak’s multiple comparison test).

To establish the catalytic-dependent and catalytic-independent requirements of Tet1 in mESC proliferation, we examined the proliferation of our previously generated [6] Tet1 knockout (*Tet1*^−/−^), Tet1 catalytic mutant (*Tet1^m^*^/*m*^) and wild type (*Tet1*^+/+^) mESCs over 4 d in a culture. We found that *Tet1*^−/−^, but not *Tet1^m^*^/*m*^, mESCs had a significantly slower proliferation than the wild type mESCs, with the difference being more prominent on day 4 (Figure 1A). The overexpression of either the wild type or the catalytically inactive Tet1 in *Tet1*^−/−^ mESCs rescued this growth defect (Figure 1B), suggesting that Tet1 non-catalytic functions are required for the proper proliferation of mESCs. To examine whether the reduced proliferation of *Tet1*^−/−^ mESCs was due to increased cell death, we stained *Tet1*^−/−^, *Tet1^m^*^/*m*^ and wild type mESCs with Annexin V and quantified the number of apoptotic cells by flow cytometry. Although the number of early apoptotic cells (7AAD^−^ annexin V^+^) was comparable among all three lines, the number of late apoptotic cells (7AAD^+^ annexin V^+^) was marginally increased in *Tet1*^−/−^ mESCs (~1% vs. ~0.5% in *Tet1^m^*^/*m*^ and *Tet1*^+/+^, respectively) (Figure 1C and Figure S1A). This insignificant increase in apoptosis could not explain the significantly reduced proliferation observed in *Tet1*^−/−^ mESCs. Thus, the slow growth was unlikely due to increased cell death and could involve defects in the cell cycle progression. To this end, we analyzed the cell cycle of *Tet1*^−/−^, *Tet1^m^*^/*m*^ and *Tet1*^+/+^ mESCs. First, we synchronized the mESCs by treating them with the microtubule inhibitor Nocodazole for 16 h to arrest the cells in the G2/M phase. We then released the cells to progress through the cell cycle and analyzed the percentage of cells in each phase of the cell cycle at 2, 4, 5 and 7 h post-release. We found that the *Tet1*^−/−^ mESCs exhibited an extended G1 phase (marked by the presence of significantly more cells in the G1 phase and fewer cells in the S phase) compared with the *Tet1*^+/+^ and *Tet1^m^*^/*m*^ mESCs, which had comparable cell cycle profiles. This difference was most prominent at 4 h post-release (Figure 1D–E and Figure S1B). These findings suggest that Tet1 non-catalytic functions are important for proper cell cycle progression and proliferation of mESCs.

### 3.2. Upregulation of the Cyclin-Dependent Kinase Inhibitor p21 (Cdkn1a) Is Responsible for Delayed Cell Cycle Progression and Reduced Proliferation in Tet1^−/−^ mESCs

**Figure 2:**
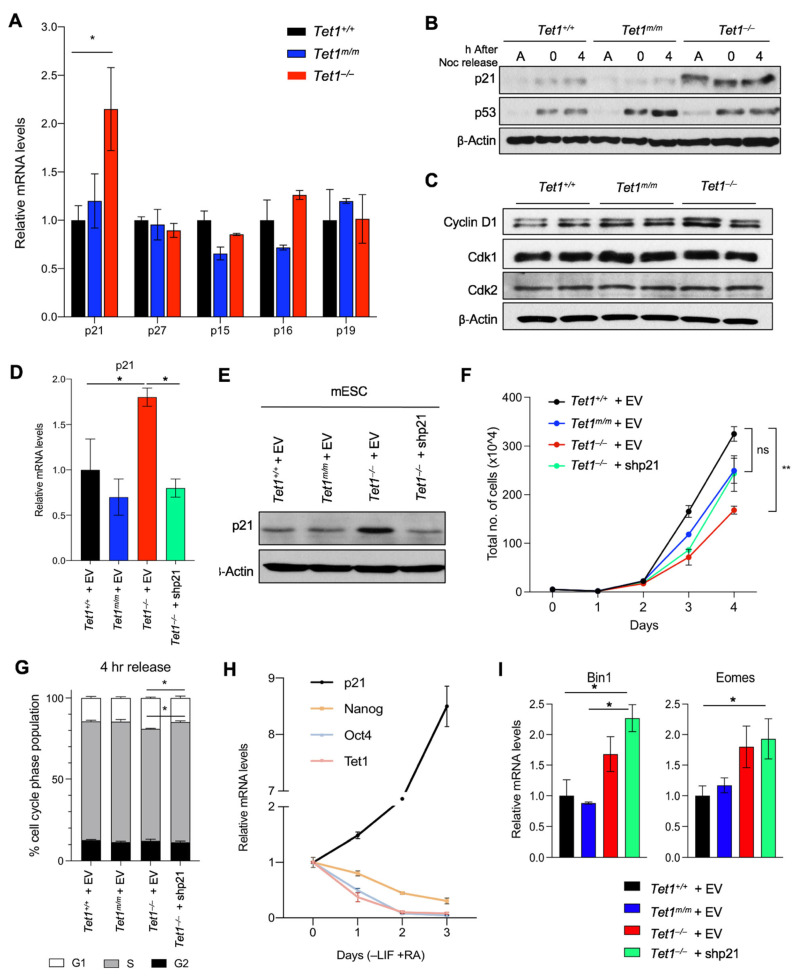
Aberrant upregulation of p21 is responsible for the slow proliferation of *Tet1*^−/−^ mESCs. (**A**) Quantification of *p21, p27, p15, p16* and *p19* mRNA levels in mESCs of indicated genotypes by RT-qPCR. Data normalized to *Gapdh* expression. Three lines of each genotype were used. (**B**) Quantification of p21 and p53 protein levels in asynchronous (**A**), G2/M-arrested (0 h) and released (4 h) mESCs of indicated genotypes by Western blot analysis. β-actin was used as a loading control. (**C**) Quantification of Cdk1, Cdk2 and Cyclin D1 protein levels in asynchronous mESCs of indicated genotypes by Western blot. β-actin was used as a loading control. (**D**,**E**) Quantification of *p21* mRNA levels by RT-qPCR (**D**) and protein levels by Western blot (**E**) in *Tet1*^+/+^, *Tet1^m^*^/*m*^, *Tet1*^−/−^ mESCs transduced with empty vector (EV) or an shRNA against p21 vector (shp21). (**F**) Growth curve of mESCs transduced with an empty vector (EV) or an shRNA against *p21* vector (shp21) over a 4-day period. (**G**) The percentage of mESCs of indicated genotypes expressing empty vector (EV) or an shRNA against *p21* (shp21) in each phase of the cell cycle 4 h after release from Nocodazole block. (**H**) Quantification of mRNA levels of *p21*, *Tet1* and pluripotency markers (Oct4 and Nanog) by RT-qPCR in wild type mESCs differentiated (−LIF +RA) for three days. Three independent mESC lines were used. (**I**) Quantification of mRNA levels of mesoderm markers *Bin1* and *Eomes* by RT-qPCR in *Tet1*^+/+^, *Tet1^m^*^/*m*^ and *Tet1*^−/−^ mESCs expressing empty vector (EV) or an shRNA against *p21* (shp21) and cultured in mESC media. Three replicates of each genotype were used. In all panels, data are presented as ± SEM; statistically significant ** *p* < 0.01 and * *p* < 0.05 (two-way ANOVA test).

To gain a molecular insight into how Tet1 regulates the ESC cell cycle, we examined our previously published transcriptomic data of *Tet1*^−/−^, *Tet1^m^*^/*m*^ and *Tet1*^+/+^ mESCs [6] where genes uniquely deregulated in *Tet1*^−/−^ mESCs were enriched for the cell cycle and proliferation gene ontology (GO) terms (Figure S2A). Among these genes, the cyclin-dependent kinase inhibitor (CKI) p21/*Cdkn1a*, which inhibits Cdk2/4 to block the G1/S transition, was a top hit that was significantly upregulated in *Tet1*^−/−^, but not *Tet1^m^*^/*m*^ and *Tet1*^+/+^, ESCs. We validated the p21 upregulation at the mRNA level by RT-qPCR (Figure 2A) and at the protein level in both asynchronous and synchronized cultures by a Western blot (Figure 2B and Figure S2B). The levels of other CKIs involved in the G1/S transition—such as p15, p16, p19 and p27—were unaffected (Figure 2A). Likewise, cyclin-dependent kinases Cdk1 and Cdk2 as well as Cyclin D were not deregulated in *Tet1*^−/−^ mESCs (Figure 2C and Figure S2B). This suggested that the upregulation of p21 is a very specific molecular signature of *Tet1*^−/−^ mESCs involved in cell cycle regulation. We found that the levels of p53, the main transcriptional activator of p21, were unaffected in *Tet1*^−/−^ ESCs (Figure 2B and Figure S2B–C). Likewise, the levels of the mir290-95 cluster, which has previously been implicated in the regulation of p21 in ESCs [24,25,26], was unaffected in *Tet1*^−/−^ mESCs (Figure S2C). Together, these findings suggested that the upregulation of p21 in *Tet1*^−/−^ mESCs is unlikely due to the perturbation of p53 or mir290-95 levels and could involve other transcriptional mechanisms involving Tet1.

Next, we sought to determine whether the p21 upregulation was specifically responsible for the proliferation of and cell cycle defects observed in *Tet1*^−/−^ mESCs. To this end, we used shRNAs to knock down p21 in *Tet1*^−/−^ mESCs equivalent to the levels in *Tet1^m^*^/*m*^ and *Tet1*^+/+^ mESCs (Figure 2D–E) and assessed the effects on proliferation and cell cycle progression. We found that reducing the p21 levels in *Tet1*^−/−^ mESCs to near to the wild type levels rescued both the slower proliferation (Figure 2F) and the elongated G1 phase (Figure 2G). This confirmed that the loss of the Tet1-mediated upregulation of p21 was driving the proliferation and cell cycle defects in *Tet1*^−/−^ mESCs. It also suggested that Tet1 was responsible for repressing p21 in the mESCs to allow for a rapid proliferation and cell cycle progression that was unique to the pluripotent state. We consistently found that during differentiation, as mESCs exited the pluripotent state (marked by the downregulation of the pluripotency factors Nanog and Oct4), the Tet1 levels decreased and the p21 levels increased (Figure 2H), corresponding with a longer G1 phase, which is a hallmark of differentiated cells. We also found that the knockdown of p21, which corrected the proliferation and cell cycle progression defects of *Tet1*^−/−^ mESCs (Figure 2F–G), did not rescue the differentiation defects of *Tet1*^−/−^ mESCs (Figure 2I). *Tet1*^−/−^ mESCs expressing either an empty vector or p21 shRNA exhibited an aberrant upregulation of mesoderm marker *Bin1* and trophectoderm marker *Eomes* compared with *Tet1^m^*^/*m*^ or *Tet1*^+/+^ mESCs expressing an empty vector. This suggested that the Tet1-mediated suppression of p21 mainly regulated the mESC cell cycle progression and not the differentiation programs.

### 3.3. Tet1 Suppresses p21 Expression by Binding to Its Promoter and Facilitating PRC2 Recruitment and H3K27 Trimethylation

**Figure 3:**
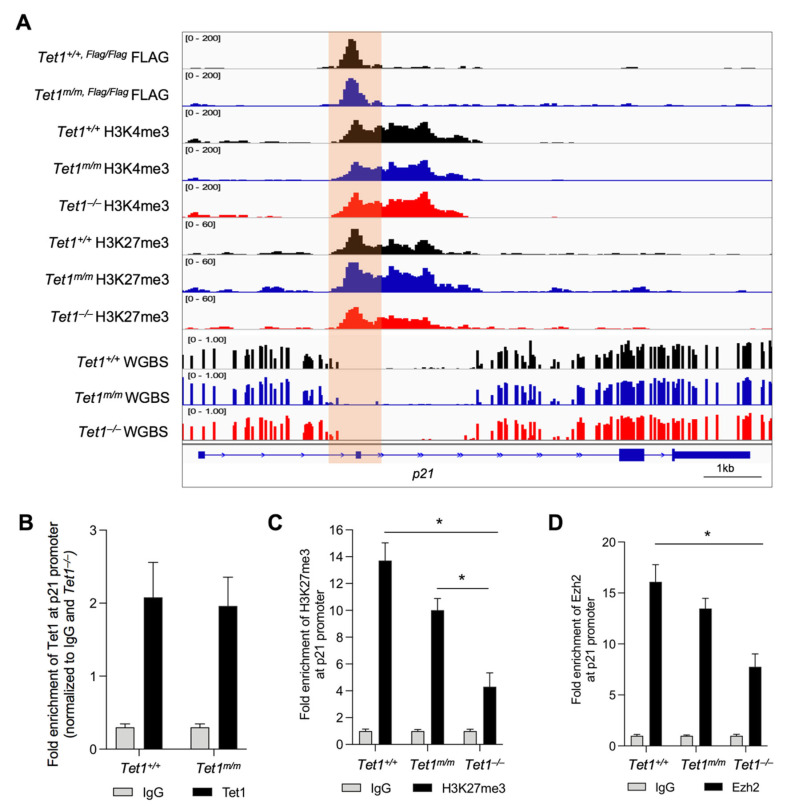
Tet1 silences p21 in mESCs by promoting Ezh2 recruitment to the p21 promoter for H3K27 trimethylation. (**A**) Genome browser tracks displaying co-occupancy of wild type and catalytic mutant Tet1 with bivalent marks (H3K4me3 and H3K27me3) and DNA hypomethylated regions at the p21 locus in mESCs of indicated genotypes using our previously published CUT&Tag and WGBS data in *Tet1*^+/+^, *Tet1^m^*^/*m*^ and *Tet1*^−/−^ mESCs (see Methods). Note that the levels of H3K27me3 are reduced in *Tet1*^−/−^ ESCs whereas DNA methylation levels are unaffected at the Tet1-bound p21 locus. (**B**–**D**) Quantification of enrichment of Tet1 (**B**), H3K27me3 (**C**) and Ezh2 (**D**) at the p21 promoter region in *Tet1*^+/+^, *Tet1^m^*^/*m*^ and *Tet1*^−/−^ ESCs (data normalized to 10% input control and IgG). Three replicates of each genotype were used. Note the significant reduction in the levels of H3K27me3 and Ezh2 at the p21 promoter in *Tet1*^−/−^ mESCs. In all panels, data are presented as ± SEM; statistically significant * *p* < 0.05 (two-way ANOVA test).

**Figure 4:**
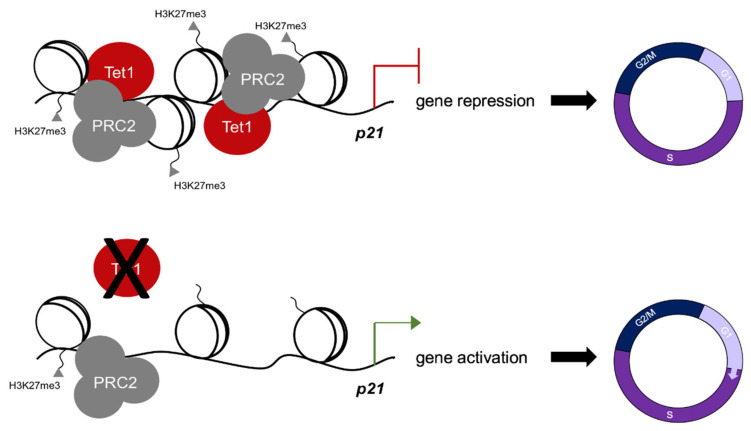
Tet1 regulates mouse ESC cell cycle. Tet1 silences p21 in mESCs by facilitating PRC2 recruitment and H3K27 trimethylation at its promoter to ensure a rapid G1/S transition unique to the pluripotent state of mESCs cultured in serum/LIF.

The upregulation of p21 in *Tet1*^−/−^ mESCs suggested that Tet1 is essential for the suppression of p21 in mESCs. Previously, we and others have implicated Tet1 in the gene suppression of mESCs where Tet1, independent of its catalytic activity, facilitates the recruitment of PRC2 to the gene promoters for H3K27 trimethylation [4,6]. This is essential for establishing the bivalency (H3K4me3^+^; H3K27me3^+^) of lineage-specific genes in mESCs, keeping them silenced in the pluripotent state and poised for activation upon differentiation [6]. This, together with the fact that p21 is a known target of PRC2 in human ESCs [12], prompted us to test whether Tet1 suppressed p21 by facilitating PRC2 recruitment and H3K27 trimethylation at its promoter. We examined the enrichment of Tet1 at the *p21* locus and assessed its co-occupancy with the PRC2 component Ezh2 and the bivalent marks using our previously published Tet1 CUT&Tag data in *Tet1*^+/+^ and *Tet1^m^*^/*m*^ ESCs as well as H3K4me3 and H3K27me3 CUT&Tag data in *Tet1*^+/+^, *Tet1^m^*^/*m*^ and *Tet1*^−/−^ ESCs [6]. Genome browser tracks revealed a significant enrichment of wild type and catalytic mutant Tet1 at the p21 promoter (Figure 3A), which was confirmed by the ChIP-qPCR (Figure 3B). The Tet1 peaks overlapped with the H3K4me3 and H3K27me3 peaks at the *p21* promoter region (Figure 3A). Although the H3K4me3 levels were comparable in *Tet1*^+/+^, *Tet1^m^*^/*m*^ and *Tet1*^−/−^ ESCs, the H3K27me3 levels were significantly reduced only in *Tet1*^−/−^ ESCs as shown in the genome browser tracks (Figure 3A) and confirmed by the ChIP-qPCR (Figure 3C). To examine if this corresponded with a reduced PRC2 occupancy, we assessed the enrichment of PRC2 component Ezh2 at the p21 promoter by ChIP-qPCR using the same primers used for assessing the H3K27me3 levels. We found that the Ezh2 levels were significantly reduced in *Tet1*^−/−^, but not in *Tet1^m^*^/*m*^*,* mESCs (Figure 3D). An assessment of the DNA methylation levels at the *p21* locus using our previously published WGBS datasets in *Tet1*^+/+^, *Tet1^m^*^/*m*^ and *Tet1*^−/−^ ESCs [6] revealed that the *p21* promoter, as with most bivalent gene promoters, was in a hypomethylated state and remained unchanged in all three lines (Figure 3A). Taken together, these data establish that in mESCs, Tet1 represses the p21 expression independent of its catalytic activity by facilitating PRC2 recruitment and H3K27me3 deposition at its promoter, which allows for the signature rapid cell cycle progression and proliferation of mESCs (Figure 4).

## 4. Discussion

Tet1 is highly expressed in mESCs where both its catalytic and non-catalytic functions have been implicated in the regulation of pluripotency and in differentiation programs [2,4,6,14,17]. However, its roles in the regulation of ESC proliferation and cell cycle progression have not been well-studied. mESCs have a unique cell cycle structure consisting of a short G1 phase that allows for a rapid cell division and proliferation [8]. This is, in part, promoted by a lack of expression of CKIs; namely, p21 [8,9]. We provided four lines of evidence that Tet1 regulates ESC proliferation by suppressing p21 independent of its catalytic activity: (1) *Tet1*^−/−^, but not *Tet1^m^*^/*m*^, ESCs had a reduced proliferation, a delayed G1-S phase progression and an increased expression of p21; (2) the re-expression of catalytic-dead Tet1 or the knockdown of p21 in *Tet1*^−/−^ ESCs rescued the proliferation defects; (3) Tet1 was enriched at the p21 promoter and its occupancy overlapped with the H3K27me3 repressive mark; and (4) a loss of Tet1, but not a loss of its catalytic activity, reduced H3K27 trimethyltransferase PRC2 occupancy as well as H3K27me3 deposition at the p21 promoter, leading to its aberrant upregulation in ESCs. These findings establish Tet1 as an epigenetic regulator of mESC proliferation in addition to its previously defined roles in mESC pluripotency and differentiation.

It is well-established that p21 expression is largely regulated by a p53-dependent mechanism at the transcriptional level in many cell types [27]. However, Tet1 loss in ESCs did not change the p53 mRNA or protein levels, implying that p21 upregulation is likely to be p53-independent unless a Tet1 deficiency perturbs p53 enrichment at the p21 promoter. Similarly, the mir290-95 cluster—which has been implicated in the regulation of p21 as well as ESC proliferation and pluripotency [24,25,26]—was not deregulated in *Tet1*^−/−^ ESCs, suggesting that p21 upregulation is not triggered by the perturbation of this cluster of miRNAs. We found that, in mESCs, p21 is a suppressed bivalent gene, marked by both repressive H3K27me3 and activating H3K4me3 histone modifications, which is consistent with studies in human ESCs where the deposition of the repressive H3K27me3 mark at the p21 promoter has been shown to override activation by p53 [12]. This generates a unique epigenetic strategy to keep certain p53 target genes poised whilst p53 maintains genomic stability in ESCs. Recently, we also showed that Tet1 promotes the establishment of the bivalency of developmental genes, keeping them silenced in mESCs, but poised for activation upon differentiation [6]. The loss of Tet1 leads to an aberrant activation of these genes and differentiation defects [6]. Here, we found that reducing the abnormally high levels of p21 in *Tet1*^−/−^ mESCs by shRNA rescued the cell cycle structure and proliferation defects, but did not correct the aberrant expression of the developmental genes. This is consistent with previous work showing that a p21 overexpression in mESCs does not cause differentiation defects [10]. Together, these suggest that the aberrant differentiation of *Tet1*^−/−^ mESCs is not caused by defects in the cell cycle. We also found that the p21 expression inversely correlated with Tet1 and the pluripotency marker expression. As the mESCs differentiated, the Tet1 levels decreased and the p21 levels increased. This allowed for the establishment of an extended G1 phase pertinent to the differentiated state as the mESCs exited pluripotency. Consistently, p21 expression has been proven to be a roadblock for reprogramming somatic cells to iPSCs and the knockdown of p21 or the selection of cells with a shorter G1 increases reprogramming efficiency [28,29,30]. Therefore, Tet1, which facilitates reprogramming by promoting pluripotency gene expression programs [31,32,33], may also play a role in silencing p21 during iPSC generation.

Our findings, implicating Tet1 in the regulation of the mESC cell cycle and proliferation, are in agreement with other studies describing a requirement for Tet1 in the proper regulation of the cell cycle in other cell types, albeit involving different mechanisms. For example, in trophoblast stem cells (TSCs), Tet1 partners with Cyclin B to stabilize its protein levels. The combined loss of Tet1 and Tet2 significantly reduced the proliferation of TSCs [34]. In NIH3T3 cells, a Tet1 loss led to the downregulation of Cyclin D1 and reduced the levels of phosphorylated RB, blocking G1/S entry [35]. We did not observe any deregulation of the key cyclins in *Tet1*^−/−^ mESCs and the p21 knockdown was sufficient to rescue the proliferation defects. Therefore, it is likely that Tet1 influences the cell cycle differently in various cell types. The Tet1-mediated regulation of p21 has also been reported in other cell types. For example, hypercholesterolemia causes Tet1 downregulation in hematopoietic stem cells and leads to the upregulation of p19, p21 and p27 by decreasing the H3K27me3 repressive mark of these genes [19]. Likewise, in human ESCs, p21 is silenced by H3K27 trimethylation at its promoter [12]. In non-small cell lung carcinomas, Tet1 acts as an oncogene and its knockdown leads to the induction of p21 as well as an increased senescence in cancer cells [36]. Finally, we note that the extended G1 phase and reduced proliferation of *Tet1*^−/−^ mESCs, although significant, was not very pronounced, suggesting that other parallel mechanisms may also control the mESC G1/S transition. In conclusion, our findings establish that Tet1, in addition to its previously known function in mESC pluripotency, is an epigenetic regulator of mESC proliferation by suppressing p21 expression. This has implications not only in ESC applications, the reprogramming of iPSCs and development, but also in various cancers where Tet1 is dysregulated.

## Data Availability

This study does not generate datasets that are archived elsewhere. All data generated are shown in this mansucript main figures and supplemental information.

## Supplementary Materials

The following are available online at https://www.mdpi.com/article/10.3390/cells11081366/s1: Figure S1: Apoptosis and cell cycle profiles of *Tet1*^+/+^*, Tet1^m^*^/*m*^ and *Tet1*^−/−^ mESCs are analyzed by flow cytometry; Figure S2: mRNA and protein levels of cell cycle regulators are quantified in *Tet1*^+/+^, *Tet1^m^*^/*m*^ and *Tet1*^−/−^ mESCs; Table S1: List of oligos used in the study.
