# Supplementary material for: Tet1 Suppresses p21 to Ensure Proper Cell Cycle Progression in Embryonic Stem Cells
Source: Cells. 2022 Apr 17;11(8):1366. doi: 10.3390/cells11081366 (PMC9025953; doi:10.3390/cells11081366)
Supplement: Supplementary file 1 [file cells-11-01366-s001.zip › cells-1653079-supplementary.pdf]

Supplementary Information

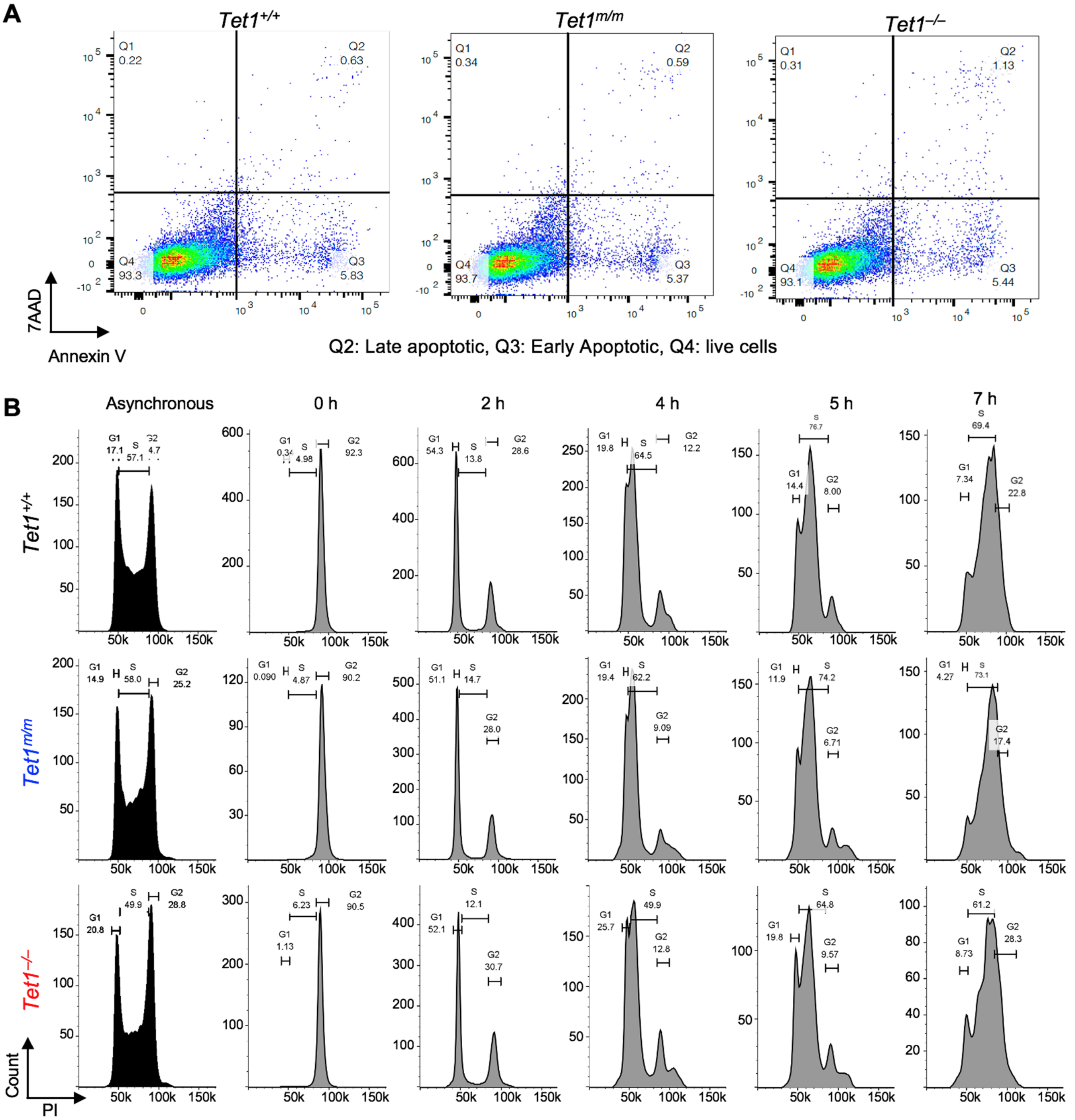

Mouse ESCs	Asynchronous (% cell population)			0 h (% cell population)			2 h (% cell population)			4 h (% cell population)			5 h (% cell population)			7 h (% cell population)		
	G1	S	G2	G1	S	G2	G1	S	G2	G1	S	G2	G1	S	G2	G1	S	G2
Tet1$^{+/+}$	17.1	57.1	24.7	0.34	4.98	92.3	54.3	13.8	28.6	19.8	64.5	12.2	14.4	76.7	8	7.34	69.4	22.8
Tet1$^{m/m}$	14.9	58	25.2	0.09	4.87	90.2	51.1	14.7	28	19.4	62.2	9.09	11.9	74.2	6.71	4.27	73.1	17.4
Tet1$^{-/-}$	20.8	49.9	28.8	1.13	6.23	90.5	52.1	12.1	30.7	25.7	49.9	12.8	19.8	64.8	9.57	8.73	61.2	28.3

Figure S1: Apoptosis and cell cycle profiles of *Tet1*$^{+/+}$, *Tet1*$^{m/m}$ and *Tet1*$^{-/-}$ mESCs are analyzed by flow cytometry. (**A**) Analysis of Annexin V and 7AAD–stained ESCs of indicated genotypes by flow

cytometry; % cells in each quadrant are shown. (**B**) Cell cycle analysis of asynchronous ESCs and synchronized (Nocodazole block for 16 h) ESCs at indicated time-points after Nocodazole release. Cells were stained by PI and subjected to a flow cytometry analysis; % cells in each phase are summarized in Table S1 below. Note that in *Tet1*$^{-/-}$ ESCs there was reduced % ESCs in the S phase and increased % ESCs in the G1 phase, particularly at 4 and 5 h post-release.

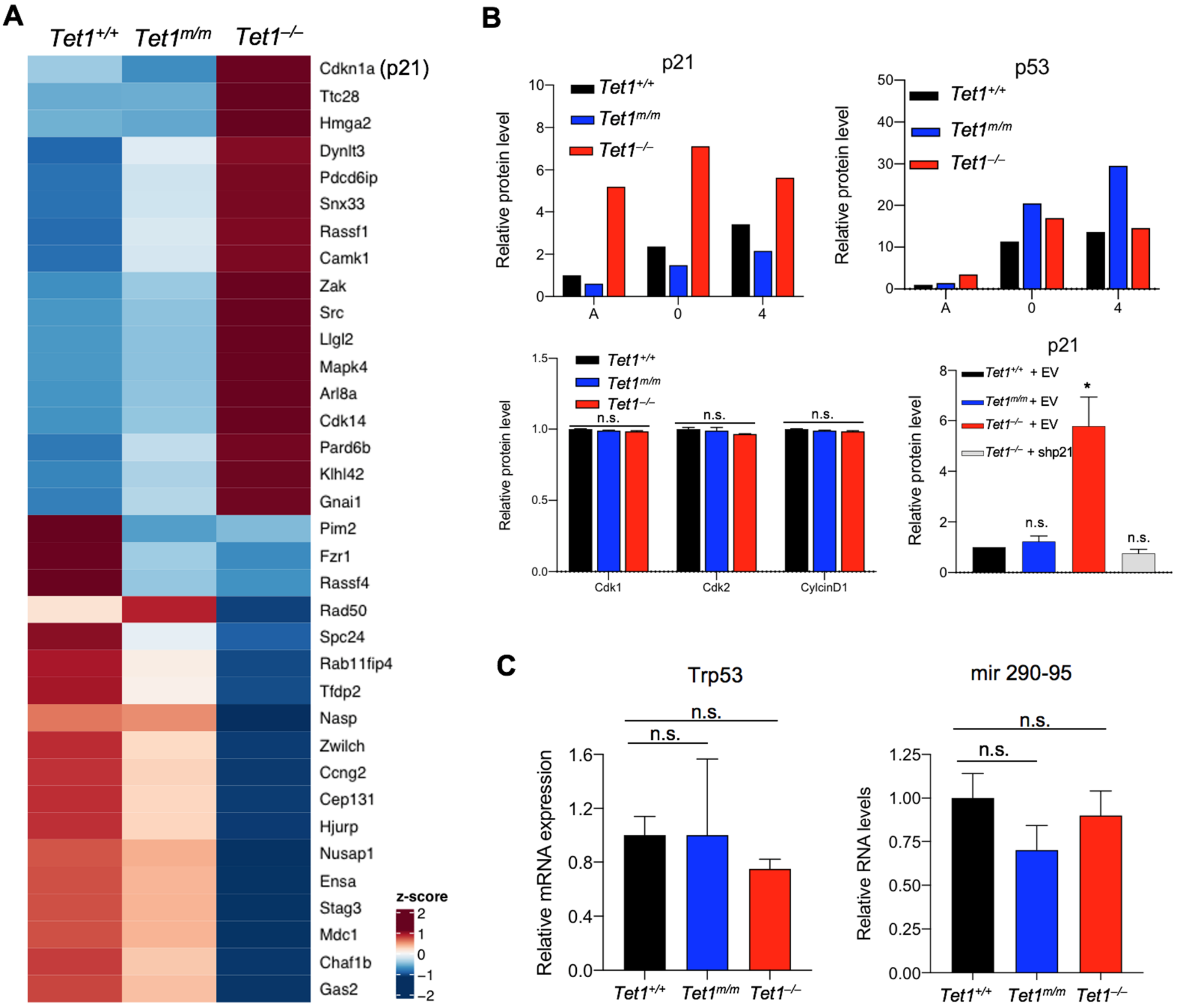

Figure S2: mRNA and protein levels of cell cycle regulators are quantified in *Tet1*$^{+/+}$, *Tet1*$^{m/m}$ and *Tet1*$^{-/-}$ mESCs. (**A**) Heatmap of the expression of genes in cell cycle regulation gene ontology terms, uniquely deregulated in *Tet1*$^{-/-}$, but not in *Tet1*$^{m/m}$ and *Tet1*$^{+/+}$; ESCs based on our published RNA-seq data (see Methods). (**B**) Quantification of protein levels of cell cycle regulators as assessed by Western blot in Figures 2B, 2C and 2E. All signals were normalized with the corresponding actin signals. (**C**) Quantification of RNA levels of p53 and pri-mir290-95 cluster in ESCs of indicated genotypes by RT-

qPCR. Data normalized to *Gapdh*. *n* = 3 ESC lines of each genotype. For all panels, error bars represent Stdev. *: statistically significant; n.s.: not significant when compared with wild type or control.

Table S1: List of oligos used in study.

Name	Sequence (5′-3′)	Purpose	Reference
Tet1 RT-qPCR For	TGCACCTACTGCAAGAATCG	Real time qPCR	[14]
Tet1 RT-qPCR Rev	AAATTGGCATCACAGCTTCC	Real time qPCR	[14]
Nanog RT-qPCR For	AAGCAGAAGATGCGGACTGT	Real time qPCR	[15]
Nanog RT-qPCR Rev	ATCTGCTGGGAGGCTGAGGTA	Real time qPCR	[15]
Pou5f1 RT-qPCR For	ACATCGCCAATCAGCTTGG	Real time qPCR	[15]
Pou5f1 RT-qPCR Rev	AGAACCATACTCGAACCACATCC	Real time qPCR	[15]
Bin1 RT-qPCR For	CAAGGCAAACTACAGGCTCATC	Real time qPCR	[16]
Bin1 RT-qPCR Rev	CCACGTTCATCTCCTCGAAC	Real time qPCR	[16]
Eomes RT-qPCR For	TGCAAGAGAAAGCGCCTGTCTC	Real time qPCR	[17]
Eomes RT-qPCR Rev	CAATCCAGCACCTTGAACGACC	Real time qPCR	[17]
Gapdh RT-qPCR For	GTGTTCCTACCCCCAATGTGT	Real time qPCR	[14]
Gapdh RT-qPCR Rev	ATTGTCATACCAGGAAATGAGCTT	Real time qPCR	[14]
p21 RT-qPCR For	CCATGAGCGCATCGCAATC	Real time qPCR	[18]
p21 RT-qPCR Rev	CCTGGTGATGTCCGACCTG	Real time qPCR	[18]
p27 RT-qPCR For	GGCCCGGTCAATCATGAA	Real time qPCR	[19]
p27 RT-qPCR Rev	TTGCGCTGACTCG CTTCTTC	Real time qPCR	[19]
p15 RT-qPCR For	GGTGGGTGCAGTCAGTACCT	Real time qPCR	[19]
p15 RT-qPCR Rev	CGAGCTGGAGGTGACTTCTC	Real time qPCR	[19]
p16 RT-qPCR For	CAACGCCCCGAACTCTTTC	Real time qPCR	[19]
p16 RT-qPCR Rev	GCAGAAGAGCTGCTACGTGAAC	Real time qPCR	[19]
p19 RT-qPCR For	CGGTATCCACTATGCTTCTGGAA	Real time qPCR	[19]
p19 RT-qPCR Rev	CCGCTGCGCCACTCAA	Real time qPCR	[19]
Trp53 RT-qPCR For	TGCTCACCCTGGCTAAAGTT	Real time qPCR	[20]
Trp53 RT-qPCR Rev	GTCCATGCAGTGAGGTGATG	Real time qPCR	[20]
Pri-mir290-95 RT-qPCR For	GAACCTCACGGGAAGTGACC	Real time qPCR	[21]
Pri-mir290-95 RT-qPCR Rev	TGCCCACAGGAGAGACTCAA	Real time qPCR	[21]
p21 For	GGCCGACGCTATAAGGAGG	Tet1 ChIP-qPCR	This paper
p21 Rev	CTATTGTTCCCTGCCACGAAG	Tet1 ChIP-qPCR	This paper
p21 For	GTGTAGGAAGGTGACCAGGC	Ezh2/H3K27me3 ChIP-qPCR	This paper
p21 Rev	CCCCAAATGCCAAACCCAAG	Ezh2/H3K27me3 ChIP-qPCR	This paper
